# In Vivo Evaluation of Nose-to-Brain Delivery of Liposomal Donepezil, Memantine, and BACE-1 siRNA for Alzheimer’s Disease Therapy

**DOI:** 10.3390/ijms251910357

**Published:** 2024-09-26

**Authors:** David Lee, Andrew M. Shen, Milin Shah, Olga B. Garbuzenko, Tamara Minko

**Affiliations:** 1 Department of Pharmaceutics, Ernest Mario School of Pharmacy, Rutgers, The State University of New Jersey, 160 Frelinghuysen Road, Piscataway, NJ 08854, USA; 2 Rutgers Cancer Institute of New Jersey, New Brunswick, NJ 08903, USA; 3 Environmental and Occupational Health Science Institute, Piscataway, NJ 08854, USA

**Keywords:** nose-to-brain delivery, Alzheimer’s disease, triple-drug therapy, liposomal delivery to brain, siRNA targeted to BACE-1, neurodegenerative disorder

## Abstract

Our study took an innovative approach by evaluating, in vivo, the efficacy of intranasal (IN) administration of liposomal formulations of donepezil, memantine, and beta-site amyloid precursor protein-cleaving enzyme (BACE-1) siRNA, and their combination as a “triple-drug therapy” in treating Alzheimer’s disease (AD). Female APP/PS1 homozygous, transgenic mice were used as an AD model. The spatial short-term memory of the APP/PS1 mice was evaluated by a Y-maze behavioral test. IN-administered formulations demonstrated better short-term memory recovery than oral administration. Triple-drug therapy induced short-term memory recovery and lowered beta-amyloid (Aβ) 40 and 42 peptide levels and BACE-1 mRNA expression. Additionally, inflammatory cytokine mRNA expression was downregulated. This innovative approach opens new possibilities for Alzheimer’s disease treatment and nose-to-brain delivery.

## 1. Introduction

Alzheimer’s disease (AD) is a slowly progressing neurodegenerative disorder with a multifactorial etiology. Suspected causes include cholinergic defects, accumulation of neurotoxic substances, oxidative stress, neuroinflammation, and mitochondrial dysfunction [1]. The traditional hypothesis implicates the aggregation of the amyloid beta (Aβ) protein in the brain, leading to downstream events such as tau hyperphosphorylation, neuroinflammation, and synaptic dysfunction [2]. The beta-site amyloid precursor protein-cleaving enzyme 1 (BACE-1 or β-secretase) and γ-secretase are responsible for a sequential enzymatic process that cleaves the amyloid precursor protein (APP), a transmembrane protein, into Aβ. The synthesis of neurotoxic Aβ peptides is primarily regulated by BACE-1 activity, which is responsible for initiating this biochemical cascade’s critical and rate-limiting phase [3]. The loss of neurons, especially in late-stage AD, highlights the urgent need for effective interventions.

Despite decades of research, the available pharmacological interventions for AD are still limited. The most-prescribed medications for AD management are donepezil, an acetylcholinesterase inhibitor, and memantine, an N-methyl-D-aspartate receptor (NMDA) inhibitor [4,5,6]. These medications are also available in combination tablets for the treatment of moderate to severe AD. Their aim is to prevent excess glutamate and degradation of acetylcholine in the brain. However, due to their lack of brain specificity, these drugs may cause systemic side effects such as nausea, diarrhea, and tremors.

In addition to small molecule-based approaches, small interfering RNAs (siRNAs) are considered potential therapeutic agents for neurological disorders. These nucleic acids have the advantage of directly inhibiting the expression of disease-associated genes with high specificity, minimal effective dosages, and a relatively simple drug-development method [7]. Studies have shown that systemic administration of BACE-1 siRNA into mouse models can reduce AD-related neuropathological features [8,9]. However, a significant challenge with siRNA delivery is that siRNAs are easily degraded by endonucleases and RNases, demonstrating poor cellular internalization, necessitating a carrier to protect and transfer these nucleic acids effectively [10,11,12]. Additionally, drug off-targeting effects can be problematic if the siRNAs do not accurately target the intended site of action [13].

The delivery of therapeutic agents to the brain is hindered by several obstacles, with one of the main barriers being the blood–brain barrier (BBB). The BBB blocks 98% of therapeutic small molecules from reaching the brain and expels these molecules through efflux pumps. The tight junctions of the BBB restrict the paracellular delivery of therapeutic agents, while transcellular transport requires specific conditions [14,15,16].

To overcome this significant challenge, the intranasal route has emerged as a potential strategy for bypassing the BBB in central nervous system (CNS) drug delivery. While historically used to induce local responses in managing allergic reactions and nasal congestions, intranasal administration is increasingly recognized for its systemic therapeutic potential. Its advantages include non-invasive application, simple administration, and bypassing of the hepatic first-pass metabolism [17,18]. Importantly, this approach provides a direct pathway to the CNS by leveraging the unique position of the olfactory mucosa outside the BBB’s scope and direct neural connections. Additionally, this method shows promise in reducing off-target drug accumulation in vital organs, potentially decreasing adverse drug side effects [19,20].

Researchers are exploring new ways to improve drug delivery to the brain. They have found nanoparticle systems, specifically liposomes, are promising for delivering drugs from the nose to the brain (NtB) [21]. Liposomes represent a highly promising carrier for NtB drug delivery due to their biocompatibility and versatile encapsulation capabilities, which accommodate both hydrophobic/hydrophilic substances and nucleic acids. They offer a controlled release of the encapsulated agents, prolong the drugs’ presence at the absorption site, and minimize systemic adverse effects [18,20,22]. Additionally, liposomes can protect siRNA from enzymatic degradation and facilitate its transport to the targeted site.

In our research, we focused on improving drug delivery to the brain. To do this, we created three liposomal formulations containing donepezil, memantine, or BACE-1 siRNA and administered them intranasally to mice to evaluate their potential therapeutic effect. Additionally, we investigated the effects of combining all three formulations, which we called a “triple liposome”.

## 2. Results

**Figure 1:**
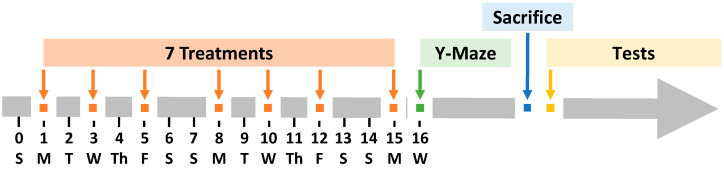
Schematic illustration of experimental timeline. APP/PS1 mice were treated seven times with various formulations within 15 days, three times per week (Monday, Wednesday, and Friday). Free donepezil and memantine were administered orally (PO) by sprinkling them in drinking water. All liposome formulations were delivered intranasally.

**Figure 2:**
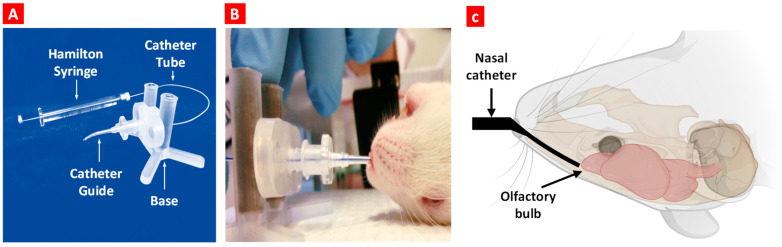
Nose-to-brain delivery in mice. (**A**) Intranasal catheter device. (**B**) The anesthetized animal is placed in the supine position, and the bent tip of the catheter is inserted in the naris. Once the catheter is inserted, the catheter tube glides into the nasal cavity until it reaches the olfactory region. (**C**) A schematic diagram shows the inserted catheter and catheter tube location before dosing.

The schematic illustration of the experimental design is presented in Figure 1. Animals were treated by oral or nose-to-brain delivery using the special intranasal catheter device shown in Figure 2.

### 2.1. Accumulation of Liposomes and siRNA in the Brain Tissue after Intravenous and Intranasal Delivery

**Figure 3:**
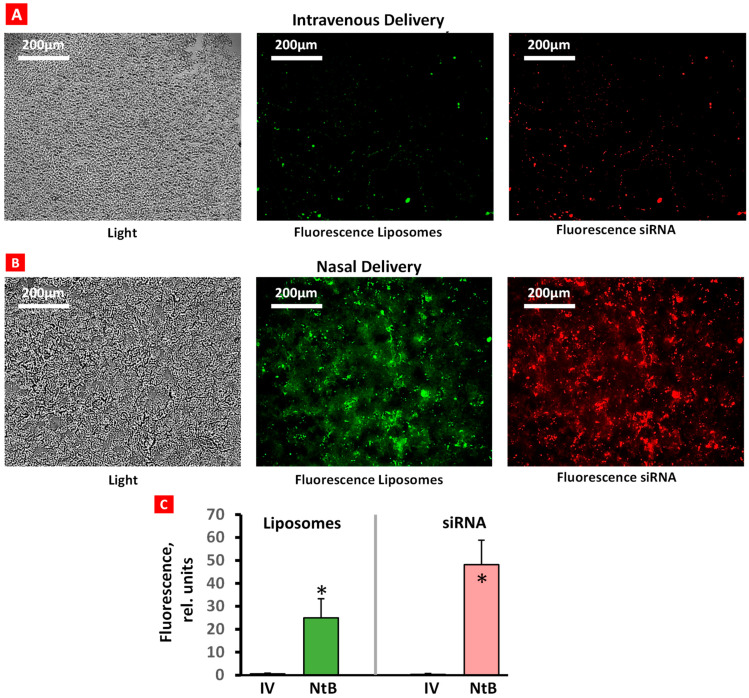
Accumulation of liposomes (green) and siRNA (red) in brain tissue after intravenous (**A**) and nasal (**B**) delivery. Representative confocal fluorescence microscope images of frozen 5 µm brain tissue sections. Average fluorescence intensity (**C**); *n* = 6, means + SD are shown. * *p* < 0.05.

The accumulation of fluorescently labeled liposomes (green fluorescence) conjugated with fluorescent siRNA (red fluorescence) in brain tissue after IN and IV delivery was evaluated using a confocal microscope (Figure 3). Only traces of liposomes and siRNA were found in brain tissues after IV injection (Figure 3A). In contrast, IN delivery resulted in a substantial accumulation of both liposomes and siRNA in the brain (Figure 3B). These data confirm the efficacy of IN delivery of liposomal siRNA formulations.

### 2.2. BACE-1, TNF-α, IL-6, IL-1β, Iba-1, and GFAP mRNA Expression in APP/PS1 Mice

**Figure 4:**
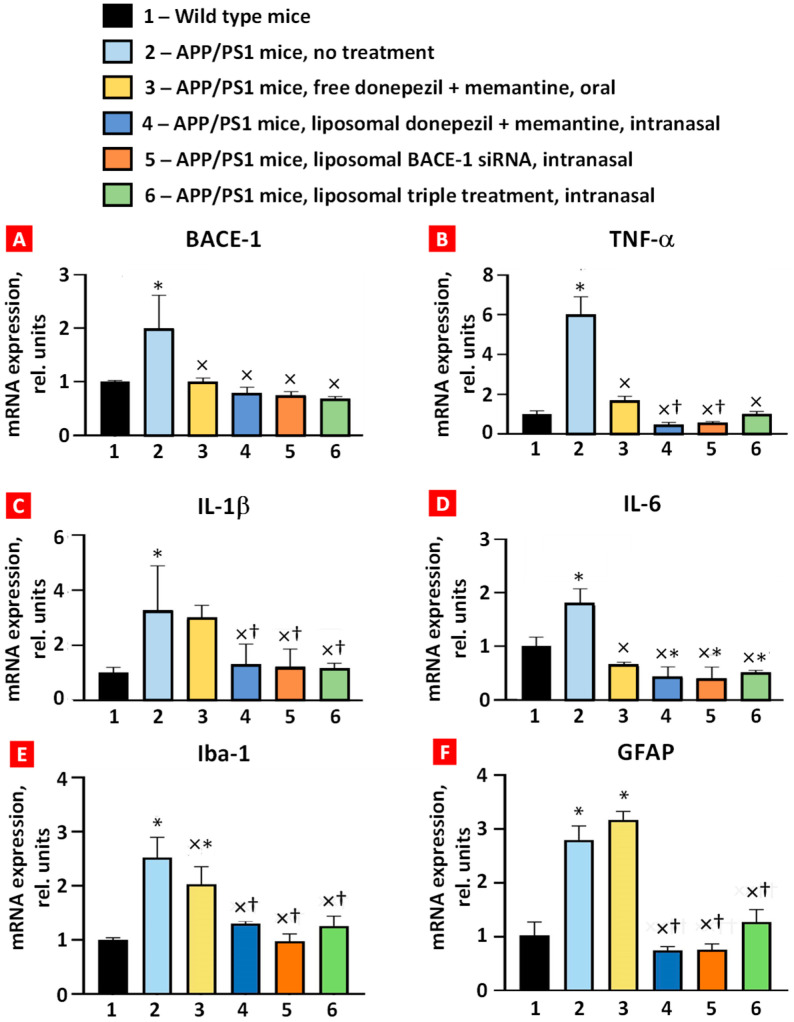
Expression of BACE-1 (**A**), TNF-α (**B**), IL-1β (**C**), IL-6 (**D**), Iba-1 (**E**), and GFAP (**F**) mRNAs in mouse brain tissues. Means ± SD are shown. * *p* < 0.01 when compared with wild-type mice (bar 1); ^×^ *p* < 0.05, when compared with APP/PS1 mice, no treatment (bar 2); ^†^ *p* < 0.05, when compared with APP/PS1 mice, free donepezil + memantine, and oral (bar 3) (*n* = 3).

The expression of BACE-1, TNF-α, IL-6, IL-1β, Iba-1, and GFAP mRNAs in whole brain was measured using real-time reverse transcription PCR (Figure 4). All six studied mRNAs were overexpressed in the brain tissues of APP/PS1 transgenic mice, which was statistically significant compared with wild-type mice. Combined oral treatment of these mice with free, non-bound donepezil and memantine decreased the expression level of BACE-1, TNF-α, IL-6, and Iba-1 mRNAs. In contrast, the expression of IL-1β and GFAP mRNA was not statistically significant. Intranasal brain delivery of combined liposomal forms of donepezil and memantine significantly decreased the expression of all studied mRNAs (Figure 4, bar 4) when compared with the untreated APP/PS1 group (Figure 4, bar 2). Moreover, the suppression of TNF-α, IL-1β, Iba-1, and GFAP was statistically more pronounced when compared with oral administration of free drugs (compare bars 3 and 4 in Figure 4). Nasal delivery of the liposomal form of BACE-1 mRNA and triple liposomal formulation statistically significantly decreased the expression of BACE-1 mRNA, TNF-α, both interleukins, Iba-1, and GFAP. Interestingly, IL-6 mRNA expression of all the intranasal treatment groups was lower than in the wild-type mice (*p* < 0.05). These data demonstrate the advantages of intranasal treatment over oral treatment in reducing inflammatory cytokine mRNA expression in the brain.

### 2.3. Soluble and Insoluble Aβ40 and Aβ42 Peptides

**Figure 5:**
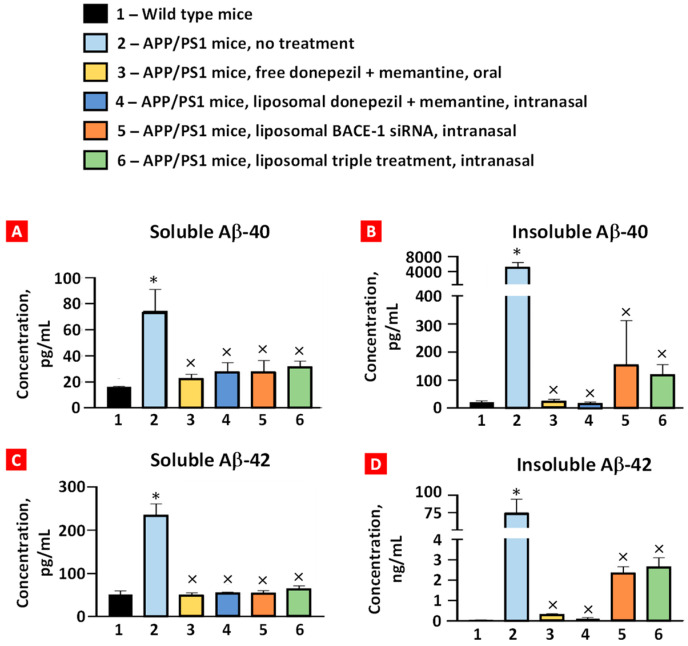
The concentration of soluble (**A**) and insoluble (**B)** Aβ40 and soluble (**C**) and insoluble (**D**) Aβ42 peptides in brain tissues. Please note that the ordinate scale in (**D**) has been changed to ng/mL. Means ± SD are shown. * *p* < 0.05 when compared with wild-type mice (bar 1); ^×^ *p* < 0.05 when compared with APP/PS1 mice, no treatment (bar 2) (*n* = 3).

The levels of soluble/insoluble Aβ40/42 peptides in the brain tissues were examined using ELISA (Figure 5). APP/PS1 mice displayed a marked statistically significant increase in soluble and insoluble forms of both peptides compared with the corresponding levels in the wild-type mice. All the treatment groups had significantly decreased levels of these peptides compared with the corresponding levels in the APP/PS1 mice. Although the average concentrations of soluble/insoluble Aβ40/42 peptides after oral and intranasal treatments with donepezil and memantine were lower than after intranasal treatment with liposomal BACE-1 mRNA and triple liposomal formulation, the difference was not statistically significant.

### 2.4. The Y-Maze Behavior Test

**Figure 6:**
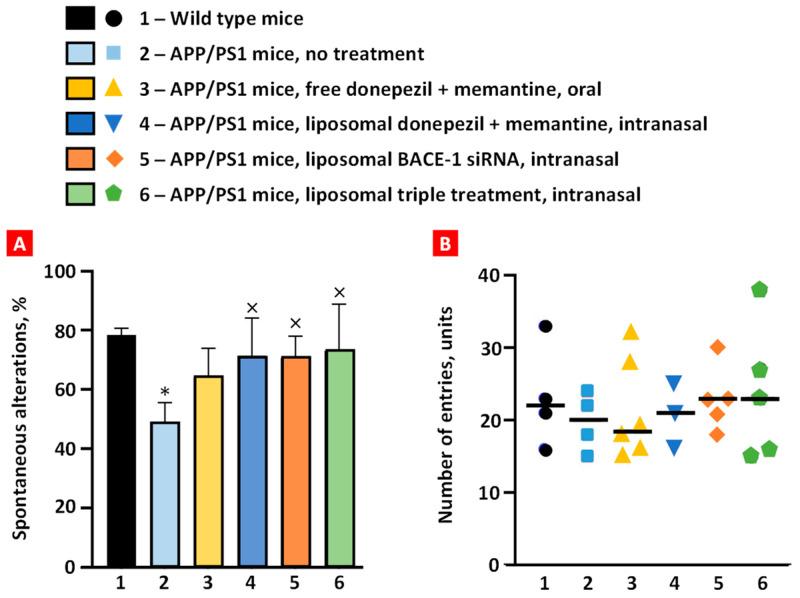
Spontaneous alternation (**A**) and number of arm entries (**B**) among the treatment groups in behavioral Y-maze test. Means ± SD spontaneous alterations are shown. * *p* < 0.05 when compared with wild-type mice (bar 1); ^×^ *p* < 0.05 when compared with APP/PS1 mice, no treatment (bar 2).

The mouse was positioned at one end and allowed 5 min to explore the three arms freely. Wild-type mice exhibited ~78% spontaneous alternation, and APP/PS1 mice demonstrated impaired short-term memory (~48%) as they showed a lower percentage of spontaneous alternation (*p* < 0.01) (Figure 6A). Spontaneous alternation of transgenic mice treated orally with free non-bound donepezil and memantine increased to 64%, but it was not statistically significant. The remaining treatment groups significantly increased the spontaneous alternation of transgenic mice to values similar to the healthy mice group (*p* < 0.05). This result demonstrates that intranasal brain administration is more effective than per os (PO) administration in enhancing short-term memory in transgenic mice.

An evaluation of arm entries among treatment groups was performed, and it demonstrated no notable variances between the groups, with the average number of arm entries being around 20 units (Figure 6B). Furthermore, intra-group variability was observed, which indicates the inherent unpredictability within each group.

## 3. Discussion

Alzheimer’s disease is a complex neurodegenerative disorder affecting cognitive, behavioral, and physical function. Toxic Aβ peptide accumulation is one of the most notable pathologic events in AD progression. This peptide arises from sequential cleavages of amyloid precursor protein (APP) by β-secretase (BACE-1), making BACE-1 a crucial therapeutic target. This is also validated as most AD patients exhibit elevated BACE-1 levels [23]. Commonly prescribed medications like donepezil and memantine alleviate symptoms, but disease-modifying options remain limited. Interestingly, recent research highlights the higher potential of combination therapies, suggesting enhanced five-year survival rates of AD patients [24]. Based on these considerations, we developed liposome formulations containing donepezil, memantine, and BACE-1 siRNA and investigated their individual and combined effects on AD treatment in vivo.

Our result of comparing intranasal and intravenous administrations demonstrated that IN delivery can efficiently increase the accumulation of active ingredients in the brain. Recent research utilizing the intravenous route has demonstrated increased blood–brain barrier (BBB) permeability in both wild-type and transgenic mice [25,26]. These studies achieved this enhancement by modifying liposome surfaces through conjugation and the incorporation of polyethylene glycol. As described earlier, nose-to-brain delivery significantly benefits brain drug delivery as it bypasses the BBB and reduces systemic exposure. The pathways for NtB delivery are not fully understood, but many recent studies have suggested three possible major pathways. One route is the direct transport of drugs to the rostral brain through olfactory nerves. Another is the direct transport of drugs to the pons through trigeminal nerves. A third possible route is indirect transport through the vasculature and lymphatic system, leading to the brain by crossing the BBB [27]. Drug absorption from NtB may not be limited by one single pathway, but several mechanisms could be combined. Thus, the higher accumulation of liposomes in the brain by NtB delivery compared with IV administration is not surprising.

Notably, the dominant pathways of NtB delivery can be determined based on the physicochemical properties of the delivery system. Li et al. formulated polycaprolactone nanoparticles and observed that the trigeminal nerve pathway was the dominant means of NtB delivery [28]. On the other hand, Kanazawa et al. formulated hydrophobic stearate oligopeptide nanoparticles, which showed that the olfactory nerve pathway was the dominant pathway [29]. Also, neutral charge liposomes are believed to diffuse more readily in the nervous system compared with charged liposomes due to their increased stability in the interstitial fluid of the perivascular and perineural cavities, thereby favoring diffusion through the NtB pathway [30,31,32]. Since the resulting overall charge of all our liposome formulations is neutral, it can be speculated that their application is better suited to NtB delivery. Further research must be done to evaluate our liposome’s dominant drug delivery pathway.

The clearance of liposomes from the brain requires further investigation to understand their potential future applications. Previous research has demonstrated that organic nanoparticles, including lipid and polymeric types, are removed from the brain primarily through the paravascular glymphatic pathway [33,34]. This pathway is a specialized clearance system mediated by astrocytes, facilitating the efficient elimination of metabolic waste from the brain. It operates through the exchange between cerebrospinal fluid and interstitial fluid, a process that is dependent on astrocytic aquaporin-4 water channels and microglia. In the context of neurodegenerative diseases such as AD, astrocytes often undergo damage due to neuroinflammation and the accumulation of amyloid beta plaques and tau tangles. This damage can impair the function of the glymphatic system, potentially affecting its ability to effectively clear nanoparticles from the brain. Therefore, it is crucial to conduct further studies to assess how impairment of the glymphatic system in neurodegenerative conditions might influence the clearance and distribution of liposomes.

In this study, we observed that donepezil, memantine, and BACE-1 siRNA liposomes can enhance learning and memory abilities in APP/PS1 mice and inhibit the accumulation of Aβ peptides as well as the mRNA expression of inflammatory cytokines such as TNF-α, IL-6, and IL-1β. Previous studies showed that donepezil, memantine, or BACE-1 siRNA as a single agent can inhibit microglial activation and decrease Aβ concentration and inflammatory cytokine release in APP/PS1 mice. Guo et al. [35] tested various concentrations of oral donepezil to evaluate TNF-α and IL-1β mRNA and protein downregulation. Although mice treated with donepezil 1mg/kg orally (per os, PO) showed a significant reduction in TNF-α and IL-1β mRNA expression, there was only a reduction in the insoluble Aβ40 group. Alley et al. [36] tested 20 mg/kg/day of memantine in drinking water for 8 days and showed a decreased concentration of Aβ peptides only in soluble Aβ42 form. Zhou et al. [37] demonstrated that BACE-1 siRNA encapsulating glycosylated nanoparticles at 1mg/kg IV significantly reduced BACE-1 mRNA and protein expression and improved short-term memory. Our study showed that a continuous dose of a combination of donepezil and memantine administered PO could decrease both the soluble and the insoluble Aβ40/42. Also, intranasal administration of liposome three times a week was as efficacious as continuous oral administration of donepezil and memantine. Since AD patients suffer from comorbidities such as hypertension and osteoarthritis and consume multiple medications, it is beneficial to have as few drug administrations as possible to minimize drug adverse events and patient error [38]. Also, intranasal administration of drugs can bypass the BBB and hepatic first-pass metabolism and increase the drug-targeting effect, allowing a decreased concentration of medication to achieve the therapeutic index. For intranasal delivery of the liposomal form of BACE-1 mRNA, the dose of siRNA was markedly reduced compared with conventional therapies (1mg/kg/day vs. 100 µg/kg/day) and still showed BACE-1 mRNA, inflammatory cytokine, and Aβ downregulation. Initial pilot studies in our lab already indicate the ability to encapsulate BACE-1 mRNA in liposomes at a higher concentration. All these results further demonstrate the added benefits of intranasal administration over oral or IV administration.

Our liposomal combinational or triple-drug therapy significantly reduced the concentration of soluble/insoluble Aβ40/42 peptides, and decreased BACE-1 mRNA and inflammatory cytokine expression in the brain. However, our study did not show a significant reduction in p-tau expression, considered another hallmark of AD, compared to untreated mice. It is plausible that beta-amyloid plaque can induce tau-phosphorylation, and decreased Aβ concentration can also downregulate p-tau expression. Previous studies have shown that BACE-1 siRNA can decrease p-tau protein expression in the cortex and hippocampus of mice brains [8,37]. One possible reason for this difference is that the downregulation of p-tau may be prominent in specific brain regions. This was not a parameter measured or followed within our study as the whole brain was digested without bias. Also, while the dose of BACE-1 siRNA was sufficient to downregulate Aβ peptides and inflammatory cytokines, it may require a higher dose to decrease p-tau expression. Subregional biochemical analysis of the brain or an increased dose of BACE-1 siRNA may be necessary to observe a decrease in tau protein.

The synergistic effect of the “triple-drug cocktail” was not significantly noticeable compared to the combination of donepezil and memantine or BACE-1 siRNA liposomes. This could be attributed to the small volume of a mouse’s nostrils. Typically, a treatment volume of 20–40 µL per nostril is administered, but delivering the three liposomes in a single administration can be challenging [39]. Notably, the volume of the triple-drug therapy could easily exceed 40 µL if given at the same time, which may necessitate different times of administration on the same day.

This behavioral study suggests that intranasal administration of liposomes is more effective than oral administration. Although the soluble/insoluble Aβ40 and 42 peptide concentrations from mice treated with free non-bound donepezil plus memantine PO were lower than triple liposomal IN treatment, the behavior test exhibited the inverse when considering short-term memory improvement. This result suggests that multifactorial causes other than Aβ peptide accumulation impair short-term memory in AD patients. One example was that all intranasal treatment formulations downregulated inflammatory cytokine mRNA expression, whereas PO drug administration did not. Also, intranasal delivery systems lowered the markers for microgliosis (Iba-1) and astrogliosis (GFAP) significantly compared with PO formulation, which suggests that microglia and astrocyte activations may affect short-term memory recovery. Further research is needed to investigate how these specific biomarkers interact and contribute to cognitive improvement through donepezil, memantine, and BACE-1 siRNA.

## 4. Materials and Methods

### 4.1. Animals

Seven- to nine-month-old female APP/PS1 homozygous, transgenic mice with C57BL/6; C3H genetic background, and wild-type (WT) mice were purchased from Mutant Mouse Resource & Research Centers (MMRRC), Bar Harbor, ME, USA. APP/PS1 mice are double transgenic mice exhibiting chimeric mouse/human amyloid precursor protein (MO/HuAPP695swe) and a mutant human presenilin 1 (PS1-dE9). This transgene allows the mice to overproduce a human Aβ peptide. The mice were housed in standard individual ventilation cages in an animal experimental system (3 mice per cage). Mice were kept under a 12 h light/dark cycle with free access to food and water. All experimental protocols were approved by the Institutional Animal Care and Use Committee (IACUC), Rutgers, the State University of New Jersey.

### 4.2. Liposomal Formulations of Drugs and siRNA

Liposomal formulations of donepezil, memantine, and BACE-1 siRNA were prepared as previously described [40]. Briefly, egg L-α-phosphatidylcholine-Egg-PC and cholesterol (Avanti Polar Lipids, Alabaster, AL, USA) were dissolved in ethanol and transferred to a volumetric rotary evaporator flask to produce a thin lipid film. The dry lipid film was hydrated with ammonium sulfate until the constituents were fully dissolved. Then, it was dry-sonicated for 1 min, extruded, and purified by dialysis (Float-A-Lyzer MWCO 100 kD, Repligen, Waltham, MA, USA) at 4 °C overnight in saline. The next day, donepezil (Thermo Fisher Scientific, Waltham, MA, USA) or memantine (Sigma, St. Louis, MO, USA) was incorporated into the liposome via the ion-gradient method. Then it was dialyzed again with Float-A-Lyzer at 4 °C overnight in NaCl 0.9% solution for purification. For the preparation of BACE-1 siRNA liposome, Egg-PC, cholesterol, and 1,2-Dioleoyl-3-trimethylammonium propane (DOTAP, Avanti Polar Lipids, Alabaster, AL, USA) were dissolved in ethanol, transferred to a round-bottom volumetric flask, and rotated under vacuum until a thin film was formed. BACE-1 siRNA (Horizon Discovery, Lafayette, CO, USA) was dissolved in nuclease-free citric acid buffer (0.1M, pH 4), added to the flask containing a dry lipid film, and hydrated until the lipid film was fully dissolved. Then, it was dry-sonicated, extruded, and purified by dialysis at 4 °C overnight in nuclease-free saline (Growcells, Irvine, CA, USA). 

### 4.3. Accumulation of Formulations in Brain

To study the accumulation of liposomal formulations in the brain after intravenous (IV) and intranasal (IN) delivery, liposomes were labeled with fluorescein isothiocyanate (FITC) with green fluorescence (Thermo Fisher Scientific, Bridgewater, NJ, USA). siGLO Red siRNA (Dharmacon™, GE Healthcare, Pittsburgh, PA, USA) was used as a model of non-specific siRNA molecules with red fluorescence instead of BACE-1 siRNA for these experiments. Then, 24 h after a single injection, animals were sacrificed, the brain was washed in ice-cold saline, and kept frozen. The fluorescence was visualized by a Zeiss Axiostar Plus confocal microscope (New York Microscope Company, New York, NY, USA) on frozen 5 µm tissue sections.

### 4.4. Drug and siRNA Administration

Separate cohorts were used for each experiment (*n* = 5 to 6 per treatment group): (1) non-treated WT mice (*n* = 5), (2) non-treated APP/PS1 transgenic mice (*n* = 6), (3) transgenic APP/PS1 mice treated with free delivered orally (*n* = 6), (4) transgenic APP/PS1 mice treated with a combination of liposomal forms of donepezil and memantine by intranasal injection (*n* = 6), (5) transgenic APP/PS1 mice treated with liposomal form of BACE-1 siRNA by intranasal injection (*n* = 6), and (6) transgenic APP/PS1 mice treated with a triple combination of liposomal forms of donepezil, memantine, and BACE-1 siRNA by intranasal injection (*n* = 6). For oral delivery, donepezil (1 mg/kg) and memantine (1 mg/kg) were sprinkled in the drinking-water chamber. The water chamber with drugs was changed every other day. For the IN group, mice were anesthetized with isoflurane gas chamber (3.75”W × 9.00”D × 3.75”H; VetEquip, Livermore, CA, USA) for 1–2 min before each treatment dose. DL (0.5 mg/kg/nostril), ML (0.5 mg/kg/nostril), and BACEL (50 µg/kg/nostril) were administered to each nostril three times a week for 15 days, for a total of 7 doses (Monday, Wednesday, and Friday). Group 4 and 5 mice received a total of 25 µL/nostril of corresponding liposomes, and Group 6 mice received 50 µL/nostril of liposomes. All the liposome formulations were administered sequentially. Then, 24 h after the last administration, mice underwent Y-maze behavior tests and were sacrificed. Mouse brain tissue samples were subsequently collected for tests (Figure 1). Nose-to-brain delivery was performed using a nasal catheter device (Neuropharma, Seattle, WA, USA, Figure 2).

### 4.5. Reverse Transcription Real-Time Quantitative Polymerase Chain Reaction (RT-qPCR)

The brain tissues were homogenized in 1 mL of phenol/guanidine-based QIAzol lysis reagent (Qiagen, Germantown, MD, USA). RNA was quantified and normalized using a NanoDrop spectrophotometer (Thermo Fisher Scientific, Bridgewater, NJ, USA) and reverse transcribed using a High-Capacity cDNA Reverse Transcription Kit with RNase Inhibitor (Thermo Fisher Scientific, Bridgewater, NJ, USA) based on the manufacturer’s protocol. BACE-1, TNF-α, IL-1β, IL-6 mRNA, Iba-1, and GFAP expression were measured by qPCR (Applied Biosystems StepOnePlus, Thermo Fisher Scientific, Bridgewater, NJ, USA) using corresponding TaqMan^TM^ Gene Expression Assays (Thermo Fisher Scientific, Bridgewater, NJ, USA). Mouse GAPDH mRNA was used as an endogenous housekeeping gene to normalize mouse BACE-1, TNF-α, IL-1β, IL-6, Iba-1, and GFAP mRNA expression levels. The data were calculated based on the comparative C_t_ method (2^−ΔΔCt^).

### 4.6. ELISA Soluble/Insoluble Aβ40, 42 Detections

The quantification of Aβ40 and 42 was determined using an Amyloid β 40 ELISA kit and an Amyloid β 42 ELISA kit (Thermo Fisher Scientific, Bridgewater, NJ, USA). Briefly, the brain tissues were weighed and homogenized in 10 µL/mg (*v*/*w*) RIPA buffer containing protease inhibitor cocktail (Thermo Fisher Scientific, Bridgewater, NJ, USA). The homogenates were centrifuged at 13,000 rpm, 4 °C for 15 min, and the supernatant was collected. This process was repeated multiple times until no further pellet was formed. All the supernatants were collected for soluble Aβ ELISA assay. The pellets were sonicated and dissolved in 5M guanidine hydrochloride/50 mM Tris-HCl, pH 8, for 3 h at room temperature. Then, they were centrifuged at 13,000 rpm, 4 °C for 30 min, and the supernatant was collected and further diluted to 0.5 M guanidine/50 mM Tris-HCL, pH 8 for the insoluble Aβ ELISA assay. All the supernatants were stored at −80 °C until use. Proteins from each sample were normalized with a Pierce^®^ protein assay kit (Thermo Fisher Scientific, Bridgewater, NJ, USA) and were analyzed with an ELISA kit based on the manufacturer’s protocol. 

### 4.7. Y-Maze Behavior Test

The spatial short-term memory of the APP/PS1 mouse was evaluated by the Y-maze test. This test evaluates spontaneous alternation behavior in rodents, which is based on their willingness to explore a new environment. Typically, healthy rodents prefer to select a maze arm different from their previous choice [41,42].

The Y-maze, a key component of our methodology, was designed with three equal arms, each measuring 35 cm in length, 5 cm in width, and 20 cm in height. These arms were attached at 120-degree angles, creating a maze-like structure (Maze Engineers, Skokie, IL, USA). The mouse was placed at one end and given 5 min to explore the three arms freely. The sequence and total number of arm entries were recorded to calculate the percent alternation. This calculation was crucial as it quantitatively measured the mouse’s spatial short-term memory. A spontaneous alternation was defined as an arm choice that differed from the last two choices. Spontaneous alternation occurred when the mouse entered a different arm of the maze in each of the three consecutive arm entries. An arm entry was counted when all four mouse paws were in the arm. Any mouse that made fewer than 15 entries was excluded. The maze was cleaned with a 70% ethanol solution between animals to eliminate odor traces. The spontaneous alternation percentage was calculated using the following equation [43,44]:

$$Spontaneous\;Alternation\left(\%\right)=\frac{Number\;of\;alternation}{Total\;number\;of\;arm\;entries\;minus\;2}\times 100$$

### 4.8. Statistical Analysis

All data are presented as the mean ± SD. All the data were analyzed by one-way ANOVA, followed by Dunnett post hoc analysis for multiple comparison tests. The level of statistical significance was set at *p* < 0.05 using GraphPad Prism V. 9.5.1 (GraphPad Software, Boston, MA, USA). 

## 5. Conclusions

In summary, our research has led to the development of liposomal formulations of donepezil, memantine, and BACE-1 siRNA for nose-to-brain delivery that effectively alleviate AD-like disorder in APP/PS1 transgenic mice. These findings highlight the potential of nose-to-brain delivery of proposed drug formulation for AD therapy and its versatility for a range of CNS diseases. This includes other neurodegenerative conditions and, possibly, brain cancer, where our liposomal formulation could be used to deliver small molecules and nucleic acids, opening new possibilities for potent therapy in these areas.

## Data Availability

This experimental material is available upon request to interested researchers.

## Abbreviations

AD, Alzheimer’s disease; APP, amyloid precursor protein; APP, amyloid precursor protein; Aβ, amyloid beta; BACE-1, beta-site amyloid precursor protein-cleaving enzyme 1; BBB, blood–brain barrier; CNS, central nervous system; DOTAP, 1,2-Dioleoyl-3-trimethylammonium propane; Egg-PC, egg L-α-phosphatidylcholine; GFAP, glial fibrillary acidic protein; Iba-1, ionized calcium-binding adapter molecule 1; IL-1β, interleukin-1β; IL-6, interleukin-6; IN, intranasal; IV, intravenous; NtB, nose to the brain; NMDA, N-methyl-D-aspartate receptor; PO, per os, oral administration; qPCR, quantitative polymerase chain reaction; RT, reverse transcription; TNF-α, tumor necrosis factor-α; WT, wild-type.
